# Bone Marrow Microenvironment as a Source of New Drug Targets for the Treatment of Acute Myeloid Leukaemia

**DOI:** 10.3390/ijms24010563

**Published:** 2022-12-29

**Authors:** Kathryn A. Skelding, Daniel L. Barry, Danielle Z. Theron, Lisa F. Lincz

**Affiliations:** 1 Cancer Cell Biology Research Group, School of Biomedical Sciences and Pharmacy, College of Health Medicine and Wellbeing, The University of Newcastle, Callaghan, NSW 2308, Australia; 2 Precision Medicine Research Program, Hunter Medical Research Institute, New Lambton Heights, NSW 2305, Australia; 3 Hunter Hematology Research Group, Calvary Mater Newcastle Hospital, Waratah, NSW 2298, Australia

**Keywords:** acute myeloid leukaemia, AML, bone marrow microenvironment, drug targets, bone marrow niche

## Abstract

Acute myeloid leukaemia (AML) is a heterogeneous disease with one of the worst survival rates of all cancers. The bone marrow microenvironment is increasingly being recognised as an important mediator of AML chemoresistance and relapse, supporting leukaemia stem cell survival through interactions among stromal, haematopoietic progenitor and leukaemic cells. Traditional therapies targeting leukaemic cells have failed to improve long term survival rates, and as such, the bone marrow niche has become a promising new source of potential therapeutic targets, particularly for relapsed and refractory AML. This review briefly discusses the role of the bone marrow microenvironment in AML development and progression, and as a source of novel therapeutic targets for AML. The main focus of this review is on drugs that modulate/target this bone marrow microenvironment and have been examined in in vivo models or clinically.

## 1. Introduction

Haematopoietic stem/progenitor cells (HSPCs) are rare, self-renewing, multipotent progenitors that produce all types of blood cells via haematopoiesis. Any disruption in haematopoiesis can lead to haematological malignancies, including acute myeloid leukaemia (AML). AML is the most common acute leukaemia in adults worldwide and has the shortest 5-year survival rate of all the leukaemia subtypes [1]. AML is a heterogeneous disease and is classified based on the cytogenetic and molecular abnormalities observed in the cancer cells [2,3].

Although a variety of treatment options for AML have been introduced over the past few decades, including targeted drugs based on the genetic abnormalities identified, mortality rates have not significantly improved, particularly among elderly patients [1]. Additionally, a significant proportion of AML patients relapse, even once a complete response has been achieved. AML relapse is due to a variety of factors, including mutations in cell cycle control pathways, dysregulation of DNA damage response, and alterations in autophagy and apoptosis [4,5,6].

Initiation and progression of myeloid malignancies were initially considered to be primarily driven by the leukaemic cell. However, emerging evidence has highlighted the importance of the bone marrow niche in supporting AML disease initiation and progression [7]. While current therapies are primarily leukaemia cell-focused, alternative approaches targeting the bone marrow microenvironment are increasingly being recognised. Herein, we will provide an overview of the normal bone marrow as well as the leukaemic microenvironment and provide a summary of clinical advances in therapeutics that specifically target the bone marrow microenvironment.

## 2. The Normal Bone Marrow Microenvironment

**Figure 1:**
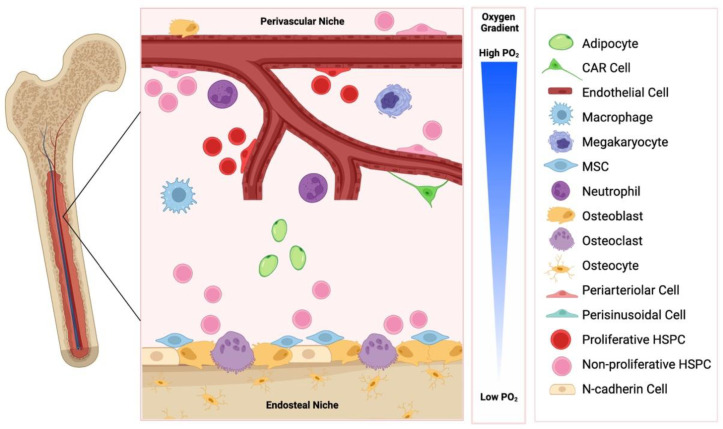
The endosteal and perivascular bone marrow niche. These niches are comprised of distinct cell types, are influence haematopoietic stem/progenitor cell (HSPC) function in a variety of ways. CAR: CXCL12 abundant reticular cells; MSC: mesenchymal stem cell. Created with BioRender.com.

The bone marrow is a heterogeneous environment comprised of various haematopoietic and non-haematopoietic cells, including osteoblasts and osteoclasts, mesenchymal stromal cells (MSCs), neurons, immune cells, adipocytes, sinusoidal endothelium and perivascular stromal cells (Figure 1). HSPCs reside in haematopoietic niches of the bone marrow, and this supportive microenvironment is essential for the long-term maintenance of a stable pool of HSPCs. HSPC functions, including proliferation, quiescence, adhesion and differentiation, are regulated by these non-haematopoietic cells through the release of variety of factors [8,9].

The haematopoietic niche is anatomically divided into two compartments: the internal endosteal bone surface and the associated perivascular network of blood vessels (Figure 1). These niches are closely related to the vascular structures, arterioles and sinusoids, respectively, and influence HSPC function in a variety of different ways [10].

### 2.1. The Endosteal Niche

The endosteal niche is defined anatomically by close proximity to cortical or trabecular bone and has a high content of osteoblasts and osteoclasts (Figure 1). In addition to their primary functions of bone remodelling, osteoblasts and osteoclasts have been implicated in regulating HSPC function, lodgement, and egress from the bone marrow. For example, osteoblastic lineage cells have been shown to control HSPC regulation in vivo via parathyroid hormone (PTH) receptor dependent signalling [11], and osteoclasts have been implicated in stem cell mobilisation in an osteopontin (OPN), stem cell factor (SCF, also known as KITL) and C-X-C motif chemokine 12 (CXCL12)/stromal cell derived factor 1 (SDF-1) mediated manner [12]. Additionally, components of the extracellular matrix, including OPN, the seven-transmembrane-spanning calcium sensing receptor (CaSR) or the sympathetic nervous system can impact HSPC function [13,14,15,16]. OPN negatively regulates HSPC numbers, and OPN^−/−^ mice exhibited increased numbers of stem cells, reduced primitive haematopoietic cell apoptosis and enhanced HSPC cycling [14,15]. Additionally, maintenance of HSPCs in a quiescent state is promoted by the interaction between angiopoietin-1 on osteoblasts and the receptor tyrosine kinase Tie2 on HSPC [17]. UDGP-galactose ceramide galactosyltransferase-deficient (*Cgt*^−/−^) mice exhibited aberrant nerve conduction and displayed no HSPC egress from the bone marrow following granulocyte colony-stimulating factor (G-CSF) or fucoidan administration, due to downregulated CXCL12 expression [16]. The Ca^2+^ content of the niche, mediated via CaSR, dictated the localisation of HSPCs, and CaSR deficient HSPCs were normal in number, proliferative and differentiation function migration and homing, however, they exhibited defective localisation, due to defective adhesion to collagen [13].

Adhesion molecules are involved in HSPC retention within the bone marrow, and as observed with CaSR deficient HSPCs, are important for directing the correct localisation of HSPCs within the bone marrow niche. While several adhesion factors have been well characterised in this function, the role of the cell adhesion molecule, N-cadherin, in HSPC lodgement into endosteal niches remains controversial. Whilst initial work demonstrated that HSPC expressed N-cadherin [18], subsequent studies have brought this into question [19]. Conditional deletion of the N-cadherin gene from osteoblasts and haematopoietic cells does not alter the frequency or the number of HSPC in the bone marrow, or their long-term or serial reconstitution potential [20]. By contrast, expression of a dominant-negative mutant of N-cadherin that inhibits both homotypic and heterotypic interactions of N-cadherin in donor HSPC reduced endosteal lodgement and compromised long-term engraftment, whereas overexpression of a wild-type N-cadherin increased endosteal lodgement and self-renewal ability [21]. Overexpression of short hairpin RNA (shRNA) specific for silencing N-cadherin gene expression in HSPC, increased HSPC proliferation, and reduced long-term engraftment and HSPC lodgement to endosteal surfaces [22]. Taken together, these studies demonstrate that the expression of N-cadherin on HSPC and its role in HSPC lodgement and function requires further clarification.

The bone marrow niche itself provides a privileged environment that supports HSPC self-renewal [23,24]. When bone marrow cells are injected into tissue engineered ectopic ossicles or in the circulation following lethal irradiation of mice to eliminate host HSPC, donor HSPCs colonised and self-renewed within the ossicles, and reconstituted haematopoiesis. This process was mediated by the proto-oncogene c-myc [24]. C-myc has been shown to control the balance between HSPC self-renewal and differentiation [25]. Conditional deletion of *c-myc* in haematopoietic cells enhanced HSPC self-renewal but inhibited differentiation and exhibited increased expression of adhesion molecules. Further, these c-myc deficient HSPC can home to and lodge into endosteal niches but failed to differentiate into mature leukocytes. Conversely, overexpression of c-myc compromised HSPC reconstitution potential following lethal BM irradiation in mouse model recipients [25]. Taken together, these studies demonstrate the importance of c-myc in normal HSPC differentiation and function and suggest that c-myc can mediate the interaction tween HSPCs and the bone marrow niche. Perhaps not surprisingly, *c-myc*, located at 8q24, is one of the most frequently activated genes in AML and overexpression plays an important role in leukaemogenesis.

In addition to supporting HSPC self-renewal, the bone marrow microenvironment supports differentiation of haematopoietic progenitor cells into various lineages through the regulation of various signalling pathways, particularly the canonical Wnt signalling pathway. β-catenin-deficient bone marrow microenvironment maintained HSPCs, but exhibited a decreased capacity to support primitive haematopoietic cells, correlated with decreased osteoblasts and production of FGF, SCF, and VCAM-1 [26]. These findings highlight the importance of the canonical Wnt signalling pathway in the bone marrow microenvironment for the maintenance of haematopoiesis.

Osteoblasts and osteoclasts have also been shown to be required for B cell development. Osteoblast ablation results in a rapid decrease in the numbers of pre-pro-B and pro-B cells [27]. In contrast, inhibition of osteoclast function results in relocalisation of B cell progenitors to the spleen [28]. Taken together, these studies suggest that the endosteal surface is required for B lymphopoiesis.

### 2.2. The Perivascular Niche

The perivascular niche is defined anatomically by close proximity to sinusoidal vascular endothelium, including surrounding supportive elements such as extracellular matrix and stromal cells (Figure 1). The blood vessels of the bone marrow are separate from the peripheral circulation [29]. It is well established that homing to the bone marrow involves an initial ‘capture’ step whereby circulating HSPC directly interact with the bone marrow endothelium. The sinusoidal endothelial cells of the bone marrow constitutively express adhesion molecules, including P-selectin, E-selectin, and vascular cell adhesion molecule-1 (VCAM-1) [30,31], which are believed to facilitate this ‘capture’ step. It is therefore unsurprising that HSPCs primarily reside in the perivascular niche in the bone marrow and spleen, however, some HSPCs are associated with endosteum [32,33]. As such, the perivascular niche is currently believed to help maintain primitive HSPCs in an undifferentiated state, and several studies have shown that the perivascular niche provides biomolecular signals that can influence HSPC function, implicating the perivascular secretome in influencing the fate of HSPCs [34].

Adventitial reticular cells, which express high levels of CXCL12/SDF-1 (CAR: CXCL12 adventitial reticular cells), and are of presumed mesenchymal origin, can alter stem cell function. Targeted deletion of CXCR4, the ligand for CXCL12/SDF-1, decreased HSPC numbers and increased sensitivity to myelotoxic injury, without impairing expansion of the more mature progenitor cells [35], highlighting that the interaction between CXCR4 and CXCL12/SDF-1 is important for the maintenance of the HSPC quiescent pool. CXCL12/SDF-1^−/−^ embryos have reduced HSPC number and function, which can be overcome by enforced CXCL12/SDF-1 expression in vascular endothelial cells [36], highlighting the importance of this chemokine in this process. Other growth factors have also been implicated in HSPC function in the bone marrow. Deletion of vascular endothelial growth factor receptor 2 (VEGFR2) in adult mice blocked regeneration of bone marrow sinusoidal endothelial cells and prevented haematopoietic reconstitution [37]. Additionally, VCAM-1 and VLA-4 adhesion molecules are implicated in the localisation and adhesion of HSPCs within the bone marrow niche [38,39]. Interestingly, CXCL12 expression and HSPC retention in the bone marrow is regulated by the sympathetic nervous system, most likely by sympathetic nerve fibres that synapse on perivascular cells around a subset of blood vessels, which thereby regulate CXCL12 expression and HSPC mobilisation via circadian oscillations [16,40]. Several other signalling pathways have been implicated in the proliferation and self-renewal in vivo. For example, wingless (Wnt) signalling is activated and necessary in the bone marrow niche to limit HSPC proliferation and preserve reconstituting capacity, and Dickkopf-1 expression in osteoblast cells reduces in vivo repopulating ability and quiescence [41].

Several subsets of bone marrow cells have been implicated in supporting immune cell function (reviewed in [42,43]). CAR cells have been shown to create a niche for HSPCs and immune cells produced in the bone marrow. Structurally, CAR cells possess long processes, and HSPCs, plasma cells, natural killer cells, plasmacytoid dendritic cells, and B cell precursors have been identified to be in contact with these processes [44,45,46,47].

Another key niche component that maintains HSPCs and is primarily expressed by perivascular cells throughout the bone marrow is SCF. Whilst HSPC frequency and function were not affected by conditional deletion of *Scf* from haematopoietic cells or osteoblasts, deletion from endothelial or leptin receptor-expressing (LepR) perivascular stromal cells led to HSPC depletion [48], highlighting the importance of SCF in promoting HSPC maintenance in the perivascular niche. By contrast, conditional deletion of SCF from LepR^+^ endothelial cells led to depleted common myeloid progenitors (CMPs), common lymphoid progenitors (CLPs), granulocyte-macrophage progenitors (GMPs), megakaryocyte-erythrocyte progenitors (MEPs), pre-MEPs, and colony-forming units-erythroid (CFU-E), as well as erythroid and myeloid blood cells. Importantly, this was not a result of HSPC depletion [49]. Taken together, this reveals cellular specialisation within the perivascular niche that is perhaps mediated in a LepR-dependent manner.

An additional key physiological regulator of HSPC function within the perivascular niche are oxygen tensions. Compared to the atmospheric oxygen tension of 21%, the HSPC niche exhibits low oxygen tensions in the range of 1–6% oxygen [50], well below the 2–9% considered by some scientists to be cellular normoxia [51]. Hypoxia activates a range of molecular responses that maintain HSPCs in a quiescent and pluripotent state, while HSPCs residing in close proximity to the vascular niche actively cycle and replenish circulating cells [52].

## 3. The Leukaemic Microenvironment

In concert with leukaemogeneic events in the haematopoietic system, the bone marrow niche is converted by AML cells into an environment that favours leukaemia cell growth and development. Cumulative evidence indicates that both leukaemic and leukaemic stem cells (LSCs) can exploit endosteal and periventricular niche signals to remodel these niches to support their proliferation and self-renewal capacities and disrupt haematopoiesis.

### 3.1. Role in Leukaemogenesis and Disease Pathophysiology

**Figure 2:**
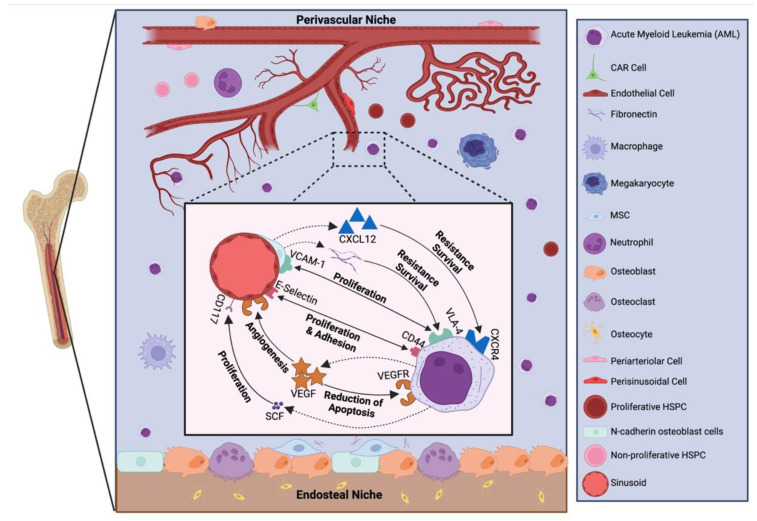
Leukaemic blast interactions with the bone marrow niche. A variety of secreted and cell–cell interactions can regulate the proliferation, survival and chemoresistance of AML cells. Many of these signalling pathways are under clinical investigation for targeted anti-AML therapies. CAR: CXCL12 abundant reticular cells; HSPC: haematopoietic stem/progenitor cell; MSC: mesenchymal stem cell; VCAM-1: vascular cell adhesion molecule 1; SCF: stem cell factor. Created with BioRender.com.

Several recent studies have provided insight into the role of aberrant signalling within the microenvironment in contributing to haematological disease pathophysiology (Figure 2). Conditional deletion of the retinoblastoma (Rb) [53] or retinoic acid receptor gamma (RARγ) [54] genes within all components of the murine haematopoietic system, led to a condition reminiscent of myeloproliferative syndromes in vivo. Similarly, Notch pathway inhibition by the deletion of the ubiquitin E3 ligase Mind bomb 1 (Mib1) under two independent promoters (*MMTV* and *Mx1*) resulted in a non-transplantable myeloproliferative neoplasm-like disease, which could be reversed by activation of Notch in the microenvironment (*MMTV-Cre;Mib1^flf^*) [55], and deletion of *Dicer1* in mesenchymal osteoprogenitors induced disordered haematopoiesis affecting multiple lineages, recapitulating key features of AML and myelodysplastic syndrome (MDS), and were microenvironment dependent [56]. Additionally, phosphatase and tensin homolog (*PTEN*) deficiency in both haematopoietic and non-haematopoietic cells resulted in myeloproliferation that progressed to overt leukaemia/lymphoma. Conversely, inducible *PTEN* deletion in haematopoietic cells in the presence of wild-type bone marrow microenvironment did not cause myeloproliferation or leukaemogenesis [57], highlighting the importance of changes within non-haematopoietic cells within the bone marrow niche in leukaemogenesis. Whilst activation of nuclear factor kappa B (NF-κB) in myelopoietic cells, and the absence of the inhibitor IκB are not sufficient for hypergranulopoiesis, these changes in the non-haematopoietic compartment resulted in increased numbers of dysplastic haematopoietic cells with progression into secondary AML [58]. Further, bone marrow stromal cell chromosomal abnormalities have also been implicated in the development of AML [59]. Taken together, these findings highlight that the interactions between leukaemic and non-haematopoietic cells within the bone marrow microenvironment may be suitable drug targets for the treatment of myeloproliferative disorders, including AML.

In addition to perturbations that occur within the bone marrow microenvironment, AML cells themselves can modify the bone marrow niche to create a ‘leukaemogeneic niche’ within the bone marrow. Further, the two-way communication between AML and endothelial cells supports AML initiation and progression. A key player identified in this is pro-angiogenic signalling is vascular endothelial growth factor (VEGF). VEGF expression and secretion was increased in AML patient bone marrow blasts compared to CD34^+^ cells cultured ex vivo from normal donors [60], consistent with the increased angiogenesis observed in bone marrow biopsies from AML patients [61]. In contrast, endosteal AML cells produce pro-inflammatory and anti-angiogenic cytokines and degrade endosteal endothelium, osteoblastic and stromal cells. These remodelled endosteal niches have reduced capacity to support non-LSCs and correlate with a loss of normal haematopoiesis [62].

Emerging evidence has demonstrated that AML cells can alter the transcriptome of surrounding bone marrow stromal cells and provide potential mechanisms for this remodelling. Gene expression profiling of bone marrow mesenchymal stromal cells isolated from mice inoculated with different human AML genotypes revealed both common and gene specific transcriptional changes in these cells. Of note, the chemoattractant SDF-1 and pathways associated with inflammation were upregulated by all subtypes. Differentially expressed genes included gene sets related to the activation of myc and those associated with mitochondria functions in mice inoculated with p53 null AML, whereas MLL/ENL inoculated mice had BM stromal cells characterised by inhibition of myc and activation of β-catenin [63]. As AML development, progression and therapy resistance involves the evolution of tumour subclones, these findings suggest that the effects of these subclones on the bone marrow microenvironment may contribute to their selection.

Another critical component of AML progression is adhesion of AML cells to the bone marrow niche. Several adhesion molecules, including, very late antigen 4 (VLA-4), E-selectin and CD44, have been implicated in AML pathogenesis (Figure 2). High expression of adhesion molecules facilitates the homing and retention of AML cells in the bone marrow niche [64,65,66,67]. The interaction between VLA-4 and VCAM-1 activates pro-survival and proliferative pathways in both AML and stromal cells via activation of the NF-κB pathway [68]. Additionally, the interaction between VLA-4 on AML cells and stromal fibronectin is crucial for the persistence of minimal residual disease in AML [65], one of the key drivers of AML relapse. E-selectin is expressed by endothelial cells and binds to CD44 expressed on AML cells. CD44 is a key regulator of AML LSCs homing to bone marrow niches and maintaining a primitive state [64]. CD44 mediates adhesion to extracellular matrix in the bone marrow niche through binding to its main ligand, hyaluronan, as well as other ligands, including OPN, fibronectin and E-selectin, all of which are involved in cell trafficking and adhesion [67,69].

Besides adhesion, AML cells can also be regulated by soluble factors secreted by cells within the bone marrow microenvironment, like CCL3, transforming growth factor beta (TGF-β) or CXCL12/SDF-1. The interaction between CXCL12/SDF-1 and its receptor CXCR4 on LSCs contributes to their homing to the bone marrow niche. AML cells initially migrate toward CXCL12^+^ vascular niches in the bone marrow [31,70]. Not surprisingly, CXCR4 expression is increased in AML patient samples [71], and the CXCL12/CXCR4 axis has also been implicated in AML cell survival, as AML cells cultured with SDF-1 activated pathways that promote survival, growth and chemoresistance [72,73]. CXCR4 expression is also associated with poor AML patient outcomes [74,75], and increased CXCR4 expression is observed in FLT3-internal tandem duplication (ITD) AML, compared with FLT3 wild-type AML [74], suggesting that the FLT3 axis may participate in the CXCR4-mediated trafficking of AML cells. Taken together, these data suggest that the SDF-1/CXCR4 axis may be a suitable therapeutic target for AML.

Emerging evidence indicates that the bone marrow microenvironment may also play a role in determining the lineage commitment of leukaemic cells. *MLL-AF9*-transduced CD34^+^ cord blood cells transplanted into immunodeficient mice could generate AML, acute lymphoblastic leukaemia (ALL) or biphenotypic leukaemia, depending on the recipient mouse strain and the presence of growth factors [76], further highlighting the influence of microenvironmental cues in LSC function. This effect on differentiation and lineage commitment appears to be bi-directional, as AML cells can also perturb MSC differentiation. Consistent with natural fat accumulation within the ageing bone marrow space [77], normal MSC exhibit signalling pathways primed toward adipocyte differentiation, in contrast to AML derived MSC which exhibit pathways associated with osteoblastic differentiation [78]. AML cells can induce osteoblastic, but inhibit adipogenic, differentiation of normal MSCs, by activating Smad1/5 signalling [79]. Additionally, AML development disrupts the sympathetic nervous system and the quiescence of MSCs, leading to an expansion of phenotypic mesenchymal stem and progenitor cells primed for osteoblastic differentiation, at the expense of HSPC-maintaining periarteriolar niche cells [80]. These alterations facilitate the induction of a pre-osteoblastic niche that enhances AML expansion.

Recent studies have demonstrated that LSCs are highly dependent on the leukaemic bone marrow microenvironment. Indeed, LSCs are immature drivers of leukaemogenesis, and are constantly evolving during AML progression and treatment [81,82]. The cross-talk between LSCs and the bone marrow microenvironment induce a supportive environment for leukaemogenesis, via secretion of chemokines, growth factors and cytokines [83]. Notably, LSCs impair normal HSPC proliferation, differentiation, and homing through secretion of a variety of cytokines and chemokines [84]. For instance, LSC secretion of IL-8 bind to CXCR2 and stimulates a variety of signalling pathways that support AML progression (including PI3K/Akt, PLC/PKC, MAPK, and NF-κB). Further, the Wnt-β-catenin pathway was required for the self-renewal of LSCs in AML mouse models in vivo [85]. Further, during leukaemogenesis, malignant clones become progressively independent of normal niche-regulated control mechanisms, which facilitates the progression of myeloid malignancies. Indeed, early in leukaemogenesis, bone marrow homing and localisation of LSCs are similar to those observed for normal HSPCs and is dependent on cell intrinsic Wnt signalling. However, as leukaemogenesis progresses, LSCs become independent of these Wnt signals, and LSC homing becomes distinct from HSPCs and is most similar to that of committed myeloid progenitors [86]. Thus, these LSCs have an immunophenotype that is more mature than HSPCs but have acquired limitless self-renewal through oncogenic transformation. Additionally, LSCs alter the bone marrow microenvironment by creating malignant areas that prevent healthy CD34^+^ cell engraftment via the release of SCF [70]. Dynamic in vivo confocal imaging of murine BM has demonstrated that both normal CD34^+^ cells as well as circulating leukaemic cells preferentially engraft into microdomains of specialised vasculature expressing E-selectin and the chemoattractant SDF-1 [31]. Once established, the leukaemia cells secrete high levels of SCF to attract and outcompete native HSPC niches for engraftment of normal CD34^+^ cells. However, the CD34^+^ cells engrafted into the malignant niche exhibited altered behaviour, their numbers declined compared to normal mice, and cytokine mediated mobilisation into the peripheral circulation was reduced in a manner similar to that described for patients with residual bone marrow disease [87].

Taken together, these studies highlight that successful treatment of leukaemia requires not only eliminating the circulating AML blasts, but also these LSCs and AML cells that are residing in the bone marrow niche. Elimination of these LSCs is important to not only induce remission, but also to prevent relapse and treatment resistance.

### 3.2. Contribution to Chemoresistance and AML Relapse

LSCs and the bone marrow microenvironment have been implicated in chemotherapeutic resistance and disease relapse. Indeed, LSCs have several intrinsic factors which contribute to chemoresistance. One of the main regulators of chemoresistance is the cell cycle status of the cell. LSCs normally reside in the bone marrow niche in a quiescent state [88], which reduces the efficacy of chemotherapeutics that target dividing cells. Further, pro-survival cell signalling pathways, including the NF-κB, Akt phosphatidylinositol-3 kinase (PI3K), Notch and Wnt-β-catenin pathways, are constitutively activated in LSCs [69,89,90], and have been further implicated in chemoresistance in LSCs.

In addition to these intrinsic chemoresistance factors in LSCs, there is mounting evidence that LSCs receive important signals from the microenvironment that regulate quiescence and chemosensitivity. Human AML CD34^+^/CD38^−^ stem cells could home to the endosteal niches of NOD/SCID/IL2rγ^null^ mice [91]. It was observed that following the homing of these cells to the endosteal niche, they became quiescent and resistant to Ara-C chemotherapy. Transplanted AML cells initially localised to the surface of osteoblasts in the epiphysial region, and 8 weeks post-transplantation, the number of leukaemia cells increased by as much as 50%. Further, after administration of high-dose Ara-C, residual leukaemia cells clustered and adhered to the blood vessels as well as to the endosteum [92], suggesting that leukaemia cells receive anti-apoptotic signals from both the osteoblasts and the vascular endothelium, and that disrupting this interaction may be a strategy for treating AML. Additionally, the interaction between the α4β1 integrin, VLA-4, and stromal fibronectin is associated with poor response to chemotherapy [65], indicating that stromal cells can influence chemosensitivity. Importantly, VLA-4-specific antibodies restored sensitivity to Ara-C.

Even though LSCs are less sensitive to chemotherapeutics than AML cells, and thereby contribute to the development of resistance, the bone marrow microenvironment can also offer a protective niche for AML cells in vivo. Co-culture of AML cells with bone marrow stromal cells provides significant protection for AML cells against chemotherapeutic-induced apoptosis [93]. This protection has been shown to be mediated through soluble factors and not just cell–cell contact. One of the main categories of soluble factors involved in this are cytokines. Co-culturing of bone marrow stromal and AML cells promotes chemoresistance via the activation of interleukin-6 (IL-6)/signal transducer and transcription activator 3 (STAT3)/oxidative phosphorylation pathways [94]. Additionally, recent evidence had identified that stromal IL-6 is the trigger for Jak/STAT3-mediated chemotherapeutic resistance [95]. A recent study identified that the increased oxidative phosphorylation and mitochondrial ATP synthesis observed in AML cells co-cultured with bone marrow stromal cells promoted chemoresistance through inhibiting 5′-adenosine monophosphate activated protein kinase (AMPK) and subsequent activation of mammalian target of rapamycin complex 1 (mTORC1). Inhibition of AMPK activation in AML cells promoted AML progression and induced chemoresistance in AML cells in vivo [96]. Further, the pro-survival factor B-cell lymphoma 2 (Bcl-2) is upregulated in AML co-cultures with stromal cells [97], and the activation of signals that can inhibit apoptosis, including Bcl-2, is correlated with poor response to chemotherapy in AML [97,98,99]. Pharmacological inhibition of these pathways may help to restore chemosensitivity in AML cells.

In addition, the bone marrow microenvironment has been implicated in facilitating a multidrug resistance phenotype. For example, several members of the adenosine triphosphate binding cassette (ABC) efflux transporters family, including the breast cancer resistance protein (BRCP) and multi-drug resistance 1 (MDR1)/P-glycoprotein, are upregulated under hypoxic conditions [100]. Further, co-culture of leukaemia cells with bone marrow-derived stromal cells altered the expression of ABC transporters MDR1, multidrug resistance associated protein 1 (MRP1), MRP2, MRP3 and BRCP in myeloid leukaemia cells in an insulin-like growth factor 1 (IGF1) signalling dependent manner [101].

AML relapse occurs as a result of persistence of these chemoresistant AML cells, as well as quiescent LSCs, which reside in the protective bone marrow niche. Following treatment cessation, AML cells can exit this protective environment, recirculate, and repopulate the host, thereby driving relapse. This highlights that targeting these AML blasts and LSCs within this niche is critical for preventing relapse. A variety of novel targets and therapeutic strategies for targeting this niche have begun to be explored.

### 3.3. The Immune Microenvironment and AML

The immunological microenvironment has also been implicated in AML development though leukaemic modifications that promote immune evasion (reviewed in [102]). AML cells can directly adapt to hide from immune recognition, as well as modify the immune cell compartments (including natural killer cells, dendritic cells, and effector T cells) to evade detection. AML cells are defective in antigen presentation, and gene expression profiling of AML blasts from relapsed patients after haematopoietic stem cell transplantation has revealed a plethora of immune-escape related perturbations, including the epigenetic downregulation of HLA class II genes, loss of HLA, and dysregulation of pathways involved in adaptive and innate immunity [103,104,105]. AML blasts aberrantly express immune checkpoint markers [103], which facilitates immune surveillance evasion. For example, AML cells express the immune checkpoint programmed-cell-death ligand-1 (PD-L1), that when recognised by the PD-1 receptor on T cells, causes T-cell exhaustion. There is a strong association between a high frequency of PD-1 and poor AML patient prognosis [106,107], suggesting that PD-1/PD-L1 inhibition may be a potential therapeutic strategy for AML. In addition to aberrant expression of immune checkpoint markers, AML cells have been shown to secrete immune inhibitory soluble factors, such as IL-10, TGF-β and indoleamine 2,3-dioxygenase 1 (IDO1) [108,109,110], which push T cell polarisation towards induced Tregs, thus promoting T cell tolerance and leukaemia progression. Further, high numbers of Tregs have been observed in patients with AML [110,111], and increased frequency of CD4^+^CD25^+^CD127^low/−^ Tregs are associated with poor AML patient prognosis [112]. Soluble factors secreted by AML cells have also been shown to modulate the microenvironment. For example, high levels of arginase II in the plasma of AML patients impaired T cell proliferation and polarised monocytes towards an immunosuppressive M2-like phenotype [113]. Additionally, VCAM-1 expression on LSCs has been implicated in aiding cancer cell escape of immune detection, and acts as a critical immune-checkpoint gate in the bone marrow via modulation of major histocompatibiilty complex (MHC) presentation on IHCs [114]. In a recent analysis of over 200,000 bone marrow cells from 40 AML patients and 3 healthy donors, it was proposed that the metabolism (namely the allocation of energy and oxygen) of AML cells can contribute to their immune evasion [115]. AML progenitor cells preferentially communicate with myeloid immune cells with an immunosuppressive phenotype. AML cells can also impact the metabolic characteristics of immune cells, and vice versa, formulating a feedback loop in the bone marrow microenvironment. This study suggests that a unique personalised therapy for AML may involve targeting unique immunometabolic profiles.

### 3.4. The Hypoxic Microenvironment and AML

Leukaemia progression in vivo has been shown to be associated with a hypoxic environment [116]. Leukaemic cells can proliferate under hypoxic conditions [116,117], suggesting that they are able to adapt to the hypoxic environment. Indeed, key regulators of hypoxia-related cellular responses, specifically hypoxia-inducible factor 1α (HIF-1α) and HIF-2α, have been implicated in leukaemogenesis and AML latency [118]. However, while HIF-1α and HIF-2α synergised to suppress AML development, they are not required for LSC maintenance [118]. Interestingly, CXCR4 expression is increased under hypoxic conditions in AML cells [119], and HIF-1α regulates CXCR4 [120]. Taken together, these data indicate that a hypoxic bone marrow microenvironment represents a conditional stem cell niche, where the CXCL12/CXCR4 axis can facilitate recruitment and retention of LSCs, and strategies that are able to target this hypoxic environment may exhibit anti-AML activity.

### 3.5. The Senescent Microenvironment and AML

The ageing haematopoietic system results in predictable clinical manifestations; including declining immunity, higher rates of anaemia and increased risk of AML/MDS [121]. At the cellular level, ageing is accompanied by increased cellular senescence, where senescent cells have reduced capacity to self-renew and are characterised by a ‘senescent associated secretory phenotype (SASP)’. The SASP includes upregulation of anti-apoptotic, pro-inflammatory and pro-fibrotic factors, and includes increased expression of Bcl-2, IL-6, CDK inhibitors p16 and p21, and intracellular accumulation of lysosomal α-galactosidase, which is commonly used as a biomarker [122]. Cell senescence can be pre-maturely induced by DNA damaging agents, such as oxidative stress [123], and although this growth arrest evolved to suppress cancer development [124], abnormal accumulation of such senescent cells is believed to contribute to pathological states associated with ageing [125]. Consistent with this theory, MSC collected from both MDS and AML patients express higher levels of p21 and are more senescent than normal MSC as evidenced by higher α-galactosidase staining [78,126]. Further studies have revealed that AML cells not only favour a senescent environment, but actively promote it through induction of reactive oxygen species via NOX2 derived superoxide [127].

Inhibition of this pathway using NOX2-knockdown AML cells resulted in less bone marrow senescence and improved survival when compared to mice engrafted with control cells, suggesting that interrupting this process in the early stages of disease may provide the most therapeutic benefit. However, cytotoxic drugs commonly used for initial treatment of AML (notably anthracyclines) can have off-target effects within the bone marrow microenvironment, creating therapy induced senescent cells that persist, impeding normal haematopoiesis and possibly contributing to disease relapse [128]. Simultaneous elimination of these cells using a new class of drugs known as ‘senolytics’, may provide a valid therapeutic strategy.

## 4. Targeting the Leukaemic Microenvironment for the Treatment of AML

As the importance of the bone marrow microenvironment in supporting LSC and AML blast growth is increasingly being recognised, it is also being regarded as a source of anti-leukaemic drug targets. Indeed, several strategies have been employed, with varying levels of success (Table 1 and Table 2).

### 4.1. Strategies to Dislodge LSCs and AML Blasts from the Bone Marrow Niche

As AML cells have been shown to create ‘sanctuary sites’ and be protected from chemotherapeutics when residing in the bone marrow niche, one potential strategy for treating AML that has been examined is disrupting the AML-bone marrow stromal cell interaction to cause cells to begin to cycle and re-enter the circulation so that they can become re-sensitive to chemotherapeutics (Table 1).

#### 4.1.1. Inhibiting CXCR4: Plerixafor and Other Antagonists

Among the adhesion molecules responsible for normal and LSC homing to bone marrow niches, CXCR4/CXCL12 axis appears to be one of the most promising potential anti-leukaemic targets under investigation and has been the subject of numerous pre-clinical and clinical studies (Table 1 and Table 2). The principal inhibitor of the CXCR4/CXCL12 axis, plerixafor (Mozobil^®^, AMD3100), is a small molecule that specifically binds to CXCR4 and inhibits its interaction with CXCL12 and subsequent downstream events including chemotaxis, which leads to rapid mobilisation of HSPCs [188]. It was originally developed as an anti-HIV drug but was soon recognised for its ability to mobilise stem cells out of the bone marrow and is currently approved by the FDA for autologous transplantation in non-Hodgkins Lymphoma and multiple myeloma [189]. Plerixafor has been further exploited for its anti-tumour properties; it increased apoptosis of AML cells in vitro, and increased survival in pre-clinical studies of AML patient derived xenograft (PDX) mouse models with high CXCR4 expression [129]. Additionally, treatment with this CXCR4 inhibitor increased the number of circulating leukaemic cells in an acute promyelocytic leukaemia (APL) mouse model, and sensitised circulating APL cells to Ara-C treatment, thereby increasing overall survival (OS), compared to chemotherapy alone [130], and decreased AML blast engraftment in bone marrow, liver and spleen in vivo [131]. Combining plerixafor with Ara-C and an anti-PD-L1 treatment significantly decreased AML blast count and prolonged survival in vivo [132]. Interestingly, post-transplant treatment with plerixafor significantly improved graft-versus-leukaemia effects in AML PDX models and promoted donor haematopoietic engraftment following allogenic haematopoietic cell transfer [133].

Due to promising pre-clinical results (Table 1), plerixafor has moved into Phase I/II clinical trials in relapsed or refractory AML (Table 2). A phase I dose-escalation trial did not find any dose-limiting toxicities, and plerixafor and G-CSF combined with busulfan and fludarabine in AML and MDS patients was shown to be safe and well tolerated, and to reduce graft versus host disease [157]. The addition of plerixafor to the myeloablative treatment for allogenic haematopoietic stem cell transplant for AML patients in their first complete remission is a safe and well-tolerated therapeutic procedure [161]. In addition to a promising safety profile, combinations of plerixafor with various chemotherapeutics have been demonstrated to exhibit clinical efficacy. For example, combination with mitoxantrone, etoposide and Ara-C, produced an overall response rate of 46% [156]. Similarly, treatment of relapsed/refractory FLT3-ITD-mutated AML patients with a combination of sorafenib, G-CSF and plerixafor resulted in a 36% response rate [162], and combination with fludarabine, idarubicin, Ara-C, and G-CSF induced a 50% response rate in primary refractory patients and 47% response rate in early relapse patients [160]. While plerixafor combined with the hypomethylating agent decitabine is well tolerated in older AML patients, and mobilisation of LSCs was observed in some patients, the clinical benefit of adding plerixafor to this regimen remains uncertain [158]. Further, due to poor patient enrollment, a Phase I clinical trial examining plerixafor and G-CSF in conjunction with timed-sequential chemotherapy was inconclusive [159]. In an attempt to resolve this uncertainty, a systematic review and meta-analysis of pre-clinical and clinical studies examining plerixafor as a treatment for AML was conducted and revealed plerixafor exhibited good pre-clinical efficacy and decreased total blast burden and increased survival compared to control animals in vivo and was well tolerated and safe in patients [190]. However, the majority of reported clinical trials were single arm studies, therefore, additional studies with larger sample sizes, controls and longer follow-up are required to conclusively determine whether plerixafor has any clinical benefit in AML.

Providing additional support for CXCR4 targeting being a valid AML treatment strategy, the high-affinity CXCR4 antagonist BL-8040 prolonged survival and reduced minimal residual disease when combined with FLT3 inhibitors in vivo [134], and the CXCR4 peptide antagonist LY2510924 induced AML cell mobilisation into the circulation and decreased tumour volume, both as a monotherapy and when combined with Ara-C and doxorubicin in an OCI-AML3 xenograft model in vivo [135]. Similarly, a fully humanised monoclonal anti-CXCR4 antibody, ulocuplumab (BMS-936564/MDX-1338) decreased tumour burden in vivo [136]. Importantly, in a Phase IIa clinical trial of relapsed/refractory patients, when BL-8040 was combined with high dose Ara-C, a composite response rate of 29% was observed [163]. Similarly, ulocuplumab and mitoxantrone, etoposide and Ara-C treatment of relapsed/refractory AML patients exhibited anti-leukaemic activity and safely improved the historic response rate achieved with the chemotherapy regimen alone [165]. Additionally, the CXCR4 peptide antagonist LY2510924, was shown to be safe and well tolerated in patients with relapsed or refractory AML in Phase I clinical trials [164]. Taken together, these studies demonstrate the effectiveness of CXCR4 targeting clinically, and suggests that the continued development of drugs targeting this axis for the treatment of AML is warranted.

#### 4.1.2. Targeting E-Selectin with Uproleselan

The endothelial cell adhesion molecule, E-selectin, is a key component of the bone marrow niche, and an important regulator of HSPC function. Additionally, AML blasts with high E-selectin expression are more likely to survive chemotherapy treatment and become drivers of relapse. Subsequently, E-selectin inhibitors are being examined as potential therapeutic strategies for the treatment of AML (Table 1 and Table 2). Indeed, inhibition of E-selectin with the small molecule mimetic uproleselan (GMI-1271), sensitises AML blasts to Ara-C chemotherapy, and significantly improves survival in a mouse model of AML compared to Ara-C alone [137]. This promising pre-clinical study highlighted that uproleselan warranted clinical investigation.

A phase I/II clinical study examining uproleselan in combination with mitoxantrone, etoposide and Ara-C for the treatment of AML has been conducted. The remission rate in relapsed and refractory AML patients was 41%, however, when used as a frontline therapy for older AML patients, the remission rate was 72% [166]. This clinical trial highlights that the addition of uproleselan to chemotherapy is not only well tolerated, but also induces high remission rates, and provides strong evidence for examination in further clinical trials.

#### 4.1.3. Targeting VLA-4

The adhesion molecule VLA-4 has been implicated in AML proliferation, chemoresistance and relapse, and also in the localisation and adhesion of HSPCs within the bone marrow niche [38,39,65,68], suggesting that targeting VLA-4 may be a viable anti-AML therapeutic strategy (Table 1). Indeed, in a mouse model of minimal residual disease, a 100% survival rate was achieved by combining VLA-4-specific antibodies or the VLA peptide inhibitor FNIII14 and Ara-C, whereas Ara-C treatment alone only slightly increased survival [65,138]. Another VLA-4 blocking agent in development is AS101, which blocks the interaction between VLA-4 and stromal fibronectin. In an AML PDX model, AS101 treatment abrogated drug resistance and prolonged survival in mice co-treated with Ara-C [139]. Taken together, these promising pre-clinical studies highlight that therapeutic targeting of VLA-4 warrants further investigation, and that clinically useful inhibitors are required.

### 4.2. Vascular Targeting and Angiogenesis as an Anti-AML Strategy

As increased angiogenesis is observed in the bone marrow of AML patients [61], anti-angiogenic therapy has been investigated as a clinical anti-AML strategy. To date, several drugs with anti-angiogenic properties have been tested in pre-clinical and clinical studies (Table 1 and Table 2).

#### 4.2.1. Targeting VEGF/VEGFR

One of the key drivers of angiogenesis within the bone marrow microenvironment is VEGF. VEGF mRNA is overexpressed in AML patient samples compared to normal controls [191], and has been suggested to play a role in AML pathogenesis. Therefore, several anti-VEGF strategies have been examined pre-clinically and clinically, with varying levels of success (Table 1 and Table 2). The anti-VEGF monoclonal antibody bevacizumab exhibits clinical activity against a variety of cancers when administered in conjunction with cytotoxic chemotherapy. When bevacizumab was combined with Ara-C and mitoxantrone in a Phase II clinical trial in refractory/relapsed AML, the overall response rate was 48%, and the complete response was 33% [167], suggesting that bevacizumab warranted additional clinical study in AML. However, additional randomised Phase II trials in elderly AML patients of bevacizumab combined with standard chemotherapy demonstrated that the addition of bevacizumab did not alter remission rates or event-free survival (EFS) but led to an unfortunate increase in severe adverse events [168]. This study demonstrated that the addition of bevacizumab to standard chemotherapy did not improve the therapeutic outcome for older AML patients and suggests that bevacizumab may not be suitable for the treatment of AML patients, particularly older cohorts.

Despite these disappointing clinical findings, other anti-VEGF/VEGFR strategies have also been examined for the treatment of AML (Table 1 and Table 2). One promising strategy that has been examined is aflibercept, the decoy VEGFR moiety with stronger affinity for VEGF than bevacizumab. Aflibercept slowed disease progression in two systemic human AML xenograft models and reduced peripheral AML burden in a primary relapsed AML model in vivo, and these effects were further enhanced when combined with doxorubicin [140]. Additionally, a novel vascular disrupting agent, combretastatin A1 (OXi4503), decreased tumour burden and improved survival in HL60 xenograft models in vivo [142], and when combined with bevacizumab, AML engraftment was reduced in both a systemic xenograft and an AML chloroma model [141]. Following on from this demonstration of pre-clinical efficacy, OXi4503 was examined in a Phase Ia trial in patients diagnosed with MDS or refractory AML, and shown to be safe and feasibly administered to patients [169]. An additional Phase Ib trial in relapsed/refractory AML patients in combination with Ara-C exhibited only a 19% overall response rate [170]. Overall, these clinical evaluations of VEGF/VEGFR targeting therapies have been largely disappointing, but additional studies with novel chemotherapeutic combinations may yield improved results.

#### 4.2.2. Blocking Angiopoietin-1/Tie2 Interactions

In addition to signaling via VEGF /VEGFR, angiopoitin-1 and -2 and their endothelial cell receptor tyrosine kinases, Tie1 and Tie2 are also important regulators of angiogenesis within the bone marrow microenvironment [192]. Primary human AML cells universally and constitutively release the Tie2 agonist, Ang-1, which can be decreased in vitro by the proteasome inhibitor bortezomib, or the IkB-kinase/NFkB inhibitor, BMS-345541 [193]. The expression of the Tie2 antagonist, Ang-2, by patient derived AML cells is more variable [194], with high circulating levels being an independent prognostic marker for better OS [195]. AML cells also express Tie2, and the angiopoietin-1/Tie2 interaction maintains LSCs in a quiescent and pro-survival state in the bone marrow niche [196]. Blocking this interaction decreases AML cell proliferation in co-cultures with microvascular endothelial cells [193]. A peptide that disrupts the angiopoietin-1/Tie2 interaction, Trebananib (AMG 386), has been widely studied in ovarian cancer [197], and due to the effects on AML cells in vitro, has begun to be examined as a potential treatment for AML (Table 2). A preliminary Phase Ib clinical trial in refractory/relapsed AML patients demonstrated that Trebananib increased plasma angiopoietin-2 levels in 7/13 patients. One AML patient exhibited a partial response, with two patients exhibiting stable disease [171]. A second arm of this study (Trebananib plus low dose Ara-C) has recently been completed (NCT01555268). In vitro results above suggest that combinations with more targeted therapies may be more effective, but whether Trebananib proves to be clinically useful in AML remains to be seen.

### 4.3. Targeting Signalling Pathways Involved in LSC Remodelling of the Bone Marrow Niche

Numerous signalling pathways have been implicated in LSC remodelling of the bone marrow microenvironment and targeting components of these niche-associated signalling pathways may present a novel strategy for increasing therapeutic effectiveness. Small molecule inhibitors targeting many of these signalling pathways have been examined clinically (Table 1 and Table 2).

#### 4.3.1. Wnt/β-Catenin Signalling

Wnt signalling constitutes a group of signal transduction pathways involved in development. Importantly, Wnt signalling is commonly upregulated in AML, and is necessary for a variety of important AML-related processes, including the maintenance of LSCs within the bone marrow niche [198], and Wnt-β-catenin signalling in the bone marrow microenvironment contributes to chemoresistance in leukaemias [199]. A variety of Wnt signalling inhibitors have been pre-clinically evaluated for the treatment of AML (Table 1).

For example, the anthraquinoneoxime-analogue BC2059 attenuates β-catenin levels, and significantly improved survival of mice xenografted with primary AML cells in vivo. This effect was synergistically enhanced when combined with the histone deacetylase inhibitor panobinostat [143]. Additionally, mice treated with a combination of the β-catenin antagonist PRI-724 and sorafenib, or the Wnt signalling inhibitors, PU-74654 or niclosamide in combination with Ara-C exhibited reduced AML burden and improved survival in vivo [144,145]. Treatment with the dual PI3K/mTOR inhibitor VS-5584 and the Wnt inhibitor ICF-001 significantly reduced AML burden and prolonged survival in vivo [146]. Similarly, oral administration of SKLB-667, a dual FLT3 and Wnt/β-catenin signalling inhibitor, decreased tumour burden in AML xenografts in vivo [147].

Based on these promising pre-clinical studies, a Phase I study of CWP2322291 (CWP291), a small molecule inhibitor of Wnt signalling, has been undertaken in AML and MDS patients. This trial demonstrated that this inhibitor was safe and exhibited single agent activity [172], highlighting that inhibitors of Wnt signalling warrant further clinical investigation.

#### 4.3.2. Therapeutic Targeting of ‘Undruggable’ myc

C-myc has been shown to control the balance between HSPC self-renewal and differentiation [25], and to play a pivotal role in leukaemia cell proliferation, apoptosis and differentiation [200]. Additionally, c-myc-dependent signalling has been shown to contribute to microenvironment-mediated drug resistance in AML. C-myc signalling was activated by MSC in AML cells and promoted AML cell survival [201]. Further, inhibition of c-myc activation overcame stroma-mediated drug resistance in both established and primary leukaemia cells [201]. As such, an inhibitor of myc has begun to be explored as a treatment for AML (Table 1). APTO-253 can bind to G4 structures in the nuclease hypersensitive element III region of the myc promoter and downregulate myc expression to trigger apoptosis in AML cell lines in vitro and primary AML patient samples ex vivo [202,203]. Despite only limited pre-clinical data being available, clinical trials examining APTO-253 in relapsed/refractory AML are underway and have shown that APTO-253 is safe and well-tolerated in these patients [173], however, whether APTO-253 also displays clinical effectiveness remains to be seen.

#### 4.3.3. Bcl-2 Pathways

The bone marrow microenvironment offers protection against a range of cytotoxic agents, and activation of anti-apoptotic signals in this microenvironment has been implicated in enhancing cell survival and resistance to therapy. Stromal cells express adhesion molecules and soluble factors [204] leading to activation of pro-survival pathways. Expression of Bcl-2, a critical pro-survival factor, is upregulated in AML co-cultures with stromal cells [97]. Importantly, Bcl-2 pathways are heterogeneously increased in AML and contribute to chemoresistance of quiescent leukaemia cells [205], suggesting that targeting this pathway may lead to the induction of apoptosis in quiescent leukaemic progenitor cells. As such, several Bcl-2 inhibitors have begun to be explored pre-clinically and clinically in AML (Table 1 and Table 2). Although primarily developed to target tumour cells, their clinical success may be due to unexpected off target effects within the bone marrow microenvironment that are only just beginning to be elucidated (as discussed in the next section). Indeed, AT-101 is a pan-Bcl-2 inhibitor and binds to the BH3 motif of the Bcl-2 family proteins to induce apoptosis in LSCs in vitro and in AML patient blasts ex vivo [206]. Additionally, combination with idarubicin in a FLT3-ITD AML patient derived xenograft model inhibited tumour growth in vivo [148]. Similarly, the BH3-mimetic Bcl-2 inhibitor, ABT-737, killed AML patient blasts and LSCs, including LSCs resistant to Ara-C and daunorubicin, ex vivo [207], and prolonged survival in vivo [149].

To overcome toxicities associated with Bcl-x_L_ inhibition, an orally bioavailable Bcl-2-selective Bcl-2 mimetic, ABT-199 (venetoclax) has been developed. Whilst venetoclax alone did not increase survival in AML xenograft models in vivo, combination with daunorubicin or gilteritinib significantly enhanced survival [150,151]. Despite limited pre-clinical evidence demonstrating efficacy as a monotherapy, venetoclax was explored as a monotherapy in a Phase II study in relapsed and refractory AML patients [174]. Modest efficacy was observed (an overall response rate of 19%), and venetoclax was shown to be safe and well tolerated in these patients. Combined with the preclinical findings, this study provided compelling evidence for examining venetoclax in combination with other chemotherapeutic strategies in relapsed and refractory AML.

Subsequently, several clinical studies have examined venetoclax in combination with various chemotherapeutics in AML patients. De-methylation agents have proven successful in treating haematological malignancies with mutations in epigenetic modifiers such as DNA methyltransferases (DNMT) and ten-eleven translocation (TET) family enzymes [208]. However, it is now recognised that these drugs may also rectify aberrant methylation and subsequent silencing of key genes that specifically occurs within the MDS/AML bone marrow microenvironment [209]. A large, multicentre, Phase Ib dose-escalation and expansion study in treatment-naïve elderly AML patients demonstrated that combining venetoclax with decitabine or azacytidine was well-tolerated and effective, as 67% of patients achieved complete remission or complete remission with incomplete count recovery [175]. Importantly, in a follow-up study of previously untreated AML patients ineligible for standard induction therapy, OS was longer, and the incidence of remission was higher among patients who received venetoclax and azacytidine, when compared to azacytidine treatment alone [176]. Similarly, combining venetoclax with the longer 10-day decitabine regimen in elderly AML patients exhibited a manageable safety profile and high efficacy, with an overall response rate of 74% being observed [177]. A prospective Phase Ib/II study evaluating fludarabine, Ara-C, G-CSF, and idarubicin combined with venetoclax in patients with newly diagnosed or relapsed/refractory AML revealed that 89% achieved a composite complete response, including 93% who were measurable residual disease negative [178]. After a median follow-up of 20 months, median event free and overall survival was not reached, and estimated 24-month survivals were 64% and 76%, respectively, which are favourable compared to historical benchmarks for induction chemotherapy. A post hoc propensity score matched analysis of 3 prospective clinical trials examining venetoclax in combination with induction chemotherapy revealed that the addition of venetoclax induced deep measurable residual disease negative remissions, and improved EFS [210]. Administering venetoclax dose ramp-up in combination with decitabine, azacitidine and low-dose cytarabine appears to be safe in patients with AML and MDS [211,212], and it is recommended for use in AML. Based on these promising studies, venetoclax in combination with azacitidine, decitabine or low-dose Ara-C has been approved for the treatment of adults with newly diagnosed AML who are ineligible for intensive chemotherapy.

##### Senolytics

AML blasts can induce a senescent phenotype in stromal cells within the bone marrow microenvironment. Importantly, these senescent stromal cells can feedback to promote AML blast survival and proliferation via the SASP [127]. These findings reveal the importance of a senescent microenvironment for AML pathophysiology, and support using senolytics as a valid therapeutic strategy for the treatment of AML. Indeed, many are already being used in human clinical trials as anti-fibrotic agents and to treat age related ailments such as macular degeneration [122]. Examples of current senolytics being trialled for such conditions include dasatinib (an FDA approved tyrosine kinase inhibitor for chronic myeloid leukaemia), the flavonoids quercetin and fistein, and importantly, the BCL-2 inhibitors, navitoclax, A1331852, and A1155463 [213]. The latter may explain the improved outcomes seen in AML patients treated with venetoclax in combination with traditional chemotherapeutics. Although designed to target the leukaemia cells, venetoclax may have the added benefit of eliminating senescent MSC cells from the bone marrow to produce a less favourable environment for persistent leukaemic blasts, and a more conducive milieu for the recommencement of normal haematopoiesis. Such double-edged swords may provide the ultimate arsenal to fight this disease.

##### 4.3.4. mTOR Pathway

The PI3K-Akt-mTOR pathway is among one of the most aberrantly upregulated pathways in AML and has been implicated in leukaemogenesis [214] and bone marrow-mediated chemoresistance [215], suggesting that it may be a suitable anti-AML therapeutic target. Despite the evidence implicating this pathway in a variety of important AML processes, the single agent activity of mTOR inhibitors in pre-clinical AML models has been modest [216]. However, clinical studies of rapamycin in a small cohort of refractory AML patients exhibited mildly cytoreductive effects [179], suggesting that higher doses and drug combinations should be the subject of future clinical trials.

Indeed, combining the rapamycin analogue, everolimus, with 1,25-dihydroxyvitamin D3 inhibited tumour growth in vivo [152]. Further, a clinical study investigating the combination of another rapamaycin analogue, temsirolimus, and lower dose clofarabine in older AML patients revealed an overall remission rate of 21% [180], suggesting that this combination may have some clinical utility. Taken together, these clinical studies (Table 2) indicate that m-TOR inhibitors warrant further clinical examination.

#### 4.3.5. Targeting NF-κB

Constitutive NF-κB expression has been observed in 40% of AML cases, and this aberrant activity has been shown to allow leukaemia cells to stimulate proliferation and evade apoptosis within the bone marrow microenvironment [217]. Additionally, reciprocal NF-κB activation in bone marrow MSCs and leukaemia cells promotes chemoresistance in AML cells [68], and pharmacological inhibition of NF-κB signalling altered the expression of genes included in a LSC signature [218], suggesting that NF-κB signalling within the bone marrow microenvironment as well as AML cells may represent an attractive target for the treatment of AML, including the potential elimination of LSCs. Several different strategies for inhibiting NF-κB signalling have been examined in AML cells (Table 1 and Table 2).

The reversible proteasomal inhibitor bortezomib can indirectly target constitutive NF-κB activation [219]. Bortezomib reduced the frequency and function of LSCs and increased OS in an MLL-AF9 AML xenograft model in vivo [153]. By contrast, when bortezomib was tested in AML patients in combination with pegylated liposomal doxorubicin or decitabine it did not improve patient outcomes [181,182]. However, combination with tipifarnib in high-risk MDS and AML patients was well tolerated, and a complete response or stable disease was observed in 6/11 (55%) of patients [183]. Additional clinical trials of bortezomib in combination with chemotherapeutics (NCT01371981, NCT01861314, NCT01420926, NCT00510939) are still ongoing, and whether these strategies exhibit clinical efficacy remain to be seen.

Another strategy for inhibiting NF-κB activity that has been examined in AML are phosphorothioate oligonucleotides that mimic the NF-κB consensus binding site. Whilst these oligonucleotides did not inhibit AML cell survival in vitro or ex vivo, they induced chemosensitivity to etoposide and Ara-C [220,221], and their effects on tumour burden or survival in vivo have not been examined.

### 4.4. Hypoxia

The bone marrow niche is a hypoxic environment where leukaemic cells preferentially reside, and hypoxia regulates AML cell proliferation and chemosensitivity [222]. This novel feature of leukemia can be exploited by an emerging group of drugs, known as hypoxia activated pro-drugs that are currently being evaluated for the treatment of AML (Table 1). These bioreductive drugs are harmless until they are selectively reduced under hypoxic conditions to form cytotoxic agents. Although developed as a means to target the hypoxic regions of solid tumours, their use is limited by clinical toxicities of thrombocytopenia and neutropenia, suggesting they may be more potent at targeting cancer cells within the bone marrow microenvironment [223].

The pro-drug Evofosfamide (TH-302), which under hypoxic conditions releases the DNA alkylating agent bromoisophosphoramide mustard, has begun to be explored as a potential AML treatment. In systemic human AML xenografts (HEL, HL60), TH-302 inhibited disease progression and increased OS, and both early and late treatment regimens were equally effective [154]. Additionally, combination with the kinase inhibitor, sorafenib, in a MOLM-13 xenograft model, synergistically enhanced the anti-leukaemic effects compared to either agent alone and prolonged survival in vivo [155].

Based on these pre-clinical findings, Evofosfamide was examined in a small Phase I study in patients with relapsed/refractory AML. Despite the promising pre-clinical results, the combined overall response rate in these patients was only 6% [184], thus suggesting that Evofosfamide may have only limited activity in these patients.

### 4.5. Immune Checkpoint Inhibitors

As AML cells can modulate the immunological microenvironment to favour leukaemogenesis [224], and the immune system has been shown to effectively target leukaemic blasts in the context of the graft-versus-leukaemia effect [225], immunomodulating agents, particularly immune checkpoint inhibitors, have been explored for the treatment of AML (Table 2). Of particular interest is the PD-L1/PD-1 interaction, as this inhibits immune responses in murine AML models, suggesting that the PD-1/PD-L1 pathway is involved in immune evasion by AML cells [226]. Further, high expression of PD-1, PD-L1 and PD-L2 in AML patients was associated with poor OS [106], providing further evidence that immune checkpoint inhibitors may be useful in the treatment of AML. Subsequently, the efficacy of several monoclonal antibodies targeting PD-1/PD-L1 have been examined as monotherapies or in combination with chemotherapeutics and hypomethylating agents.

Pidilizumab was the first PD-1 inhibitor examined in AML/MDS, and pidilizumab was shown to be relatively safe in a phase I trial in patients with advanced haematological malignancies [185]. The response in AML patients was disappointing, with only 1/8 AML patients exhibited a reduction in the number of peripheral blasts. Subsequently, no further clinical studies examining pidilizumab in the treatment of AML have been performed.

The expression of PD-L1, PD-L2, PD-1 and CTLA4 is enhanced in MDS by treatment with the DNA hypomethylating agent decitabine [227], suggesting that combining immune checkpoint inhibitors with this or similar agents may enhance efficacy. A non-randomised open-label phase II study examining the combination of the hypomethylating agent, azacitidine, and another PD-1/PD-L1 inhibitor, nivolumab, in relapsed and refractory AML patients produced an encouraging response rate and OS in these patients [186]. Additional studies of nivolumab combined with induction chemotherapy demonstrated that this regimen was feasible and safe in younger AML patients, however no significant improvement compared to a contemporary cohort examining Ara-C plus idarubicin was observed [187,228]. This disappointing outcome underscores the heterogeneity of the disease and absolute requirement for more suitable biomarkers to predict response to targeted treatments. Clinical trials examining other immune checkpoint inhibitors in combination with other chemotherapeutics or hypomethylating agents are nevertheless still being examined [229]. Whether any of these novel combinatorial strategies demonstrates clinical efficacy remains to be seen.

## 5. Conclusions

The bone marrow microenvironment is highly complex and is critical for supporting leukaemogenesis and AML progression and is a key player in the development of resistance as well as AML relapse. A variety of approaches for targeting the bone marrow microenvironment are being explored for the treatment of AML, with varying levels of success. Whilst promising results have been obtained using strategies that inhibit AML cell adhesion and homing, such as targeting the CXCR4/CXCL12 axis, VLA-4 and E-selectin (Table 1 and Table 2), with several of these agents, particularly the CXCR4 antagonists plerixafor and ulocuplumab, and the E-selectin inhibitor uproleselan, demonstrating clinical efficacy that warrants further investigation, other potential approaches targeting the bone marrow microenvironment have not yielded as encouraging results. For example, targeting angiogenesis and exploiting hypoxia with pro-drugs have all yielded disappointing results for the treatment of AML. Pre-clinical and early evidence of Wnt/β-catenin inhibitors are encouraging, and whether targeting myc will be a viable clinical option remains to be seen.

Taken together, these studies indicate that targeting aspects of the bone marrow microenvironment are a potentially novel therapeutic strategy for the treatment of AML, particularly for AML that is relapsed and refractory, which has traditionally poor patient outcomes. The recent approval of venetoclax acts as a proof of principle for this approach and highlights that this niche is a promising and emerging new area of focus for the identification of novel treatments for AML.

## Figures and Tables

**Table 1:** Summary of strategies for targeting the bone marrow microenvironment as a treatment for AML that have been examined experimentally in vivo.

Target	Drug	Treatment Design	Combination Treatments Design	Model/Disease Type	Results	Ref.
CXCR4	TN140	14 mg/kg/day for 7 days s.c.	-	AML PDX	Increased mobilization of leukaemic cells, decreased AML burden and significantly increased OS compared to PBS control	[129]
	Plerixafor (AMD3100)	20 mg/kg/day for 7 days s.c.	-	AML PDX	Increased mobilisation of leukaemic cells and decreased AML burden and non-significantly increased OS compared to PBS control	
		5 mg/kg s.c.	500 mg/kg Ara-C s.c.	APL cells from spleens of mCG^PR/+^ mice xenograft	Increased circulating leukaemia blast counts and sensitised cells to Ara-C treatment, increased OS compared to Ara-C	[130]
		2.5 mg/kg s.c.	100 mg/kg Ara-C i.p.	C1498 xenograft	Increased susceptibility of AML cells to Ara-C, and decreased blast engraftment in bone marrow, liver and spleen in vivo	[131]
		2.5 mg/kg s.c.	100 mg/kg Ara-C i.p. and/or anti-PD-L1 i.p.	C1498 xenograft	Combination of plerixafor, with Ara-C and anti-PD-L1 decreased AML blast % and prolonged OS in vivo	[132]
		5 mg/kg twice daily for 5 days s.c.	Allo-HCT	Murine primary MLL-AF9-AML xenograft	Posttransplant treatment with plerixafor significantly improved graft-versus-leukaemia effects in PDX, and promoted donor haematopoietic engraftment following allo-HCT	[133]
	BL-8040	400 μg s.c. day 1–7	AC220 10 mg/kg oral day 1–7	MV4-11, THP-1 or U937 xenografts	Combination increased OS and reduced minimal residual disease	[134]
	LY2510924	2.5 mg/kg s.c. day 1–21	Ara-C 50 mg/kg i.v. day 1–5 or doxorubicin 1.5 mg/kg i.v. day 1–3	OCI-AML3 or AML PDX	LY2510924 significantly improved OS compared to control and doxorubicin and reduced tumour burden compared to control; combination further enhanced OS	[135]
	Ulocuplumab (BMS-936564)	3–10 mg/kg i.p.	Ara-C 20–90 mg/kg	Nomo-1 and HL-60 xenografts	Decreased tumour volume compared to control	[136]
E-selectin	Uproleselan	40 mg/kg i.p.	Ara-C 100 mg/kg i.v. 5 days + doxorubicin 1 mg/kg i.v. day 1–3	Murine MLL-AF9 AML transplant	Combination significantly increased OS compared to chemotherapy alone	[137]
VLA-4	Monoclonal antibody	1–2 mg i.p.	Ara-C 20–40 mg i.p.	U937 or AML PDX	100% OS was observed in the combination group	[65]
	FNIII14	1 mg i.v.	Ara-C 20 mg i.p.	U937 xenograft	100% OS was observed in the combination group	[138]
	AS101	0.5 mg/kg i.p. 3× weekly	Ara-C 40 mg i.p. day 3, 4	AML PDX	Combination increased OS in both VLA-4+ and negative models	[139]
VEGF	Aflibercept	5–25 mg/kg s.c./i.p. twice weekly	Doxorubicin 3 mg/kg i.p.	HL60/VCR, HEL and AML PDX	Aflibercept increased OS; combination with doxorubicin decreased tumour burden	[140]
Vascular Targeting	OXi4503	10 mg/kg i.p. 3× week for 2 weeks	Bevacizumab 4 mg/kg i.p. weekly for 2 weeks	KG-1 and AML PDX	OXi4503 as a monotherapy decreased engraftment, whereas bevacizumab did not. Combination further decreased engraftment	[141]
		2.5–75 mg/kg once a week for 2 weeks	-	HL60 xenograft	Decreased tumour burden and increased survival	[142]
Wnt/β-catenin signalling	BC2059	1, 5, or 10 mg/kg i.v. 2× week for 3 wks	Panobinostat 5 mg/kg i.v. 3× week for 3 weeks	OCI-AML3 and AML PDX	Significantly improved survival as a monotherapy; Combination further significantly increased survival	[143]
	PRI-724	40 mg/kg s.c. mini pump	Sorafenib 5 or 10 mg/kg oral daily	MOLM-13 and AML PDX	PRI-724 as a monotherapy did not impact tumour burden or OS; Combination significantly decreased tumour burden and increased survival	[144]
	PU-74654	0.5 mg/kg	Ara-C 25 mg/kg i.p. for 5 days	U937 xenograft	Combination significantly increased OS and decreased tumour burden compared to control and Ara-C	[145]
	LiCl	25 mg/kg	Ara-C 25 mg/kg i.p. for 5 days	U937 xenograft	Combination significantly increased OS and decreased tumour burden compared to control and Ara-C	
	Niclosamide	10 mg/kg	Ara-C 25 mg/kg i.p. for 5 days	U937 xenograft	Combination significantly increased OS compared to control and Ara-C, but did not significantly decrease tumour burden	
	ICG-001	50 mg/kg/d i.p.	VS5584 5 mg/kg/d i.p.	MOLM-14 or OCI-AML2 xenografts	Combination decreased tumour burden and prolonged OS	[146]
	SKLB-667	1, 3, or 10 mg/kg/d oral	-	MV4-11 xenograft	Increased OS and decreased tumour burden	[147]
Bcl-2	AT-101	50 mg/kg oral days 1–10	Idarubicin 0.5 mg/kg i.v. day 1–3	AML PDX	Combination significantly decreased tumour burden compared to either agent alone	[148]
	ABT-737	75 mg/kg i.p. 3× wk	-	MRP8[NRASD12/hBCL-2] transgenic mice	Significantly increased OS	[149]
		75 mg/kg i.p. days 1–5 and 8–12	Daunorubicin 3 or 5 mg/kg i.v. days 1, 4, 9	MLL-A49 HSPC transplant	ABT-737 as a monotherapy did not alter OS; combination with daunorubicin significantly increased OS	[150]
	Venetoclax (ABT-199)	100 mg/kg oral day 1–5 and 8–12	Daunorubicin 3 or 5 mg/kg i.v. days 1, 4, 9	MLL-A49 HSPC transplant	ABT-199 as a monotherapy did not alter OS; combination with daunorubicin significantly increased OS	
		85 mg/kg oral day 1–27	Gilteritinib 40 mg/kg oral day 1–27	MV4-11 xenograft	No effect on OS as a monotherapy; Combination significantly improved OS	[151]
mTOR	Everolimus (RAD001)	3 mg/kg oral gavage every other day	1,25(OH)_2_D_3_ 0.05 mg/kg i.p 2× week	U937 xenograft	Combination inhibited tumour growth	[152]
NF-κB	Bortezomib	1 mg/kg i.p. once every 3 days	-	MLL-AF9 transformed mice and AML PDX	Decreased tumour burden and increased OS	[153]
Hypoxia	Evofosfamide (TH-302)	50 mg/kg i.p. 5× week for 3 weeks	-	HEL and HL60 xenografts	Decreased tumour burden and increased OS	[154]
		50 or 75 mg/kg i.p. 3× week for 2 weeks	Ara-C 100 mg/kg i.p. 5 days + doxorubicin 3 mg/kg i.v. 3 days or sorafenib 5 mg/kg orally daily for 2 weeks	Murine bone marrow cells expressing AML1/ETO, MOLM-13 and AML PDX	Increased OS when administered as a monotherapy; combination with Ara-C and doxorubicin or sorafenib decreased tumour burden and increased OS	[155]

AML—acute myeloid leukaemia; Ara-C—cytarabine; allo-HCT—allogenic haematopoietic cell transplant; CML—chronic myeloid leukaemia; MDS—myelodysplastic syndrome; PDX—patient derived xenografts; i.p.—intraperitoneal; s.c.—subcutaneous; ‘-‘—not examined.

**Table 2:** Summary of strategies for targeting the bone marrow microenvironment as a treatment for AML that have been examined clinically in humans.

Target	Drug	Treatment Design	Combination Treatment Design	Model/ Disease Type	Results	Ref.
CXCR4	Plerixafor (AMD3100)	0.08–0.24 mg/kg s.c.	Mitoxantrone 8 mg/m^2^/d i.v. + etoposide 100 mg/m^2^/d i.v. + Ara-C 1000 mg/m^2^/d i.v.	Phase I/II clinical in Relapsed/ Refractory AML patients; n = 52 (NCT00512252)	CR + CRi of 46%; increased mobilisation of leukaemic blasts into peripheral circulation; no evidence of symptomatic hyperleukocytosis or delayed count recovery with addition of Plerixafor	[156]
		0–240 μg/kg for 4 days	G-CSF 10μg/kg s.c. + Busulfan 130 mg/m^2^ i.v. + Fludarabine 40 mg/m^2^ i.v.	Phase I/II clinical in AML (n = 34), MDS (n = 7), CML (n = 4) patients	Compared to historical data set of patients treated with busulfan and fludarabine alone (n = 164), study patients exhibited lower rates of graft vs. host disease and no significant difference in RFS or OS	[157]
		320–810 μg/kg i.v. on days 1–5	Decitabine 20 mg/m^2^ on days 1–10 of each cycle	Phase I clinical in treatment-naïve older (≥60) AML patients (n = 69) (NCT01352650)	Overall response rate of 43%; most common toxicities were myelosuppression and infection; Plerixafor did not induce clinically significant hyperleukocytosis	[158]
		240 or 340 μg/kg/d	Daunorubicin 60 mg/m^2^/d i.v. for 3 days + Ara-C 500 mg/m^2^/d i.v. for 3 days + G-CSF 5 μg/kg/d on days 1–10	Phase I clinical in first-relapsed AML patients (n = 10) (EudraCT 2011-000474056)	Most plerixafor-related non-haematological adverse events were reversible grade 1 and 2 in severity; 1 patient exhibited grade 4 haematological toxicity at the lowest dose; 9 patients achieved CR + Cri; due to poor patient enrollment, the trials was concluded early	[159]
		120–240 g/kg i.v. day 1–4	Fludarabine 30 mg/m^2^/d i.v. day 1–4 + idarubicin 10 mg/m^2^/d i.v. day 1–3 + Ara-C 2 g/m^2^/d i.v. day 1–4 + G-CSF 5 μg/kg/d s.c. day 1–4	Phase I/II clinical in relapsed/ refractory AML (n = 57) (NCT01435343)	20/41 (49%) achieved CR + CRi; 3/41 (7%) died during induction; 13/26 (50%) primary refractory patients and 7/15 (47%) early relapsed patients achieved CR + Cri; median OS and DFS were 9.9 and 13 months, respectively	[160]
		240 μg/kg s.c.	Fludarabine 50 mg/m^2^/d for 4 days + bisulfan 3.2 mg/kg/d for 4 days followed by allo-HCT	Phase I clinical in AML patients in first remission (n = 12) (NCT01141543)	Adverse events potentially related to plerixafor were transient and not severe; main adverse events were nausea and dizziness in 4/12 (33%) patients and fatigue in 4/12 (33%) patients; 2/12 (17%) patients relapsed post-HCT and 6/12 (50%) were alive at last follow-up	[161]
		240 mcg/kg s.c. day 1–7	Sorafenib 400–800 mg twice-daily oral continuously + 10 mcg/kg s.c. day 1–7	Phase I clinical in relapsed/ refractory AML patients (n = 28)	No DLT were encountered in the 4-wk DLT window, but hand-foot syndrome and rash were seen beyond the window requiring dose reductions in most patients; 36% response rate (CR = 4/28 [14%], complete remission with incomplete platelet recovery = 4/28 [14%], CRi = 1/28 [4%], partial response = 1/28 [4%])	[162]
	BL-8040	0.5–2 mg/kg s.c. day 1,2	Ara-C 1.5–3 g/m^2^/d i.v.	Phase IIa clinical in relapsed/ refractory AML (n = 42) (NCT10838395)	CR + CRi observed in 12/42 (29%) patients; median survival was 8.4 months for all patients, 10.8 months in the 1.5 mg/kg phase and 21.8 months for responding patients in this cohort	[163]
	LY2510924	10–30 mg/d s.c. day 1–7	Idarubicin 12 mg/m^2^ i.v. 3 days + Ara-C 1.5 mg/m^2^ i.v. 4 days	Phase I clinical in relapsed/ refractory AML patients (n = 11) (NCT02652871)	1/11 (9%) patients experience DLT (grade 3 rash and myelosuppression); Overall response rate was 4/11 (36%) patients	[164]
	Ulocuplumab (BMS-936564)	0.3–10 mg/kg	Mitoxantrone 8 mg/m^2^ + etoposide 100 mg/m^2^ + Ara-C 1 g/m^2^ i.v. day1–5	Phase I clinical in relapsed/ refractory AML (n = 66)	Ulocuplumab was escalated to 10 mg/kg without any DLT; CR + CRi was 51%; patients with first complete remission > 6 months had better OS (16/23 [70%]) than those with complete remission less than 6 months or primary induction failure (6/20 [30%]); transient, mild/moderate thrombocytopenia was the only treatment related adverse event with ulocuplumab monotherapy	[165]
E-selectin	Uproleselan	5–20 mg/kg i.v. twice daily	Mitoxantrone 10 mg/m^2^ i.v. + etoposide 100 mg/m^2^ i.v. + Ara-C 1000 mg/m^2^ i.v.	Phase I/II clinical in relapsed/ refractory AML patients (n = 66) (NCT02306291)	No DLT were observed in the first 19 patients; CR + CRi of 41% was observed; median OS was 8.8 months; addition of uproleselan was associated with low rates of oral mucositis	[166]
VEGF	Bevacizumab	10 mg/kg i.v. day 8	Ara-C 2 g/m^2^ i.v. day1–3 + mitoxantrone 40 mg/m^2^ i.v. day 4	Phase II clinical in relapsed/ refractory AML patients (n = 48)	Myelosuppression occurred in all patients; toxicities included decreased ejection fraction (6%), cerebrovascular bleed (4%), mortality (15%); Overall response was 23/48 (48%) patients, with complete response observed in 16/48 (33%). Median OS and DFS for complete response patients were 16.2 months and 7 months, respectively	[167]
		5–10 mg/kg i.v. day 1, 15	Daunorubicin 45 mg/m^2^ i.v. day1–3 + Ara-C 200 mg/m^2^ i.v. day 1–7 for cycle 1; Ara-C 1000 mg/m^2^ i.v. twice daily day 1–6 for cycle 2	Phase II clinical in older AML patients (n = 171) (NRT904)	Complete remission rates (65% in both) and 12-month EFS in the 2 arms were not different (33% in standard arm vs. 30% in bevacizumab arm); the frequencies of severe adverse events were higher in the bevacizumab arm compared to the control arm, but the percentages of death or life-threatening severe adverse events were lower in the bevacizumab arm (60% vs. 75%, respectively)	[168]
Vascular Targeting	OXi4503	2.5–7.81 mg/m^2^ i.v. day 1, 8, 15, 22	-	Phase Ia clinical in relapsed/ refractory AML and MDS patients (n = 16) (NCT01085656)	Fever occurred in 7/18 (39%) patients; other side effects included bone pain in 5/18 (28%), flu-like symptoms 5/18 (28%), hypertension 5/18 (28%), thrombocytopenia 5/18 (28%); grade 3 or 4 hypertension and QT prolongation were not observed	[169]
		3.75–9.76 mg/m^2^ i.v., day1, 4	Ara-C 1 g/m^2^ i.v. day 1–5	Phase Ib clinical in relapsed/ refractory AML (n = 29) (NCT02576301)	The most common grade ¾ treatment-related adverse events were febrile neutropenia (28%), hypertension (17%), thrombocytopenia (17%) and anaemia (14%); no grade 5 adverse events were observed; drug-related serious adverse events, including febrile neutropenia, pneumonia/acute respiratory failure and hypotension developed in 4/29 (14%) patients; MTD was defined as 9.76 mg/m^2^ in combination with 1 g/m^2^ Ara-C; Overall response rate of 19% was observed; Median OS for the 4 patients who achieved a CR + CRi was 528 days, which was longer than the media OS of 113 days for the remaining 22 patients who did not achieve CR + CRi	[170]
Angiopoiten-1/ Tie2	Trebananib (AMG 386)	15 or 30 mg/kg i.v. weekly	-	Phase Ib clinical in relapsed/ refractory AML patients (n = 13) (NCT01555268, NCI-2011-02979)	1.15 mg/kg patient had a partial response; 2 patients (1.15 mg/kg, 1.30 mg/kg) exhibited stable disease >1 cycle	[171]
Wnt/ β-catenin signalling	CWP2322291 (CWP291)	4–334 mg/m^2^/d i.v. day 1–7	-	Phase I clinical in relapsed/ refractory AML (n = 64) and MDS (n = 5) patients (NCT01398462)	Most common adverse events were nausea (64%), vomiting (46%), diarrhea (36%), and infusion-related reactions (29%); grade 3 treatment-related adverse events occurred in 5% of patients, and were pneumonia, hypophosphatemia, leukocytosis, nausea, cellulitis, sepsis, hypokalemia, and hypertension; DLTs included nausea, abdominal pain, anaphylactic reaction, myalgia, and rash; MTD was defined as 257 mg/m^2^; Complete response was observed in 1/54 patients; partial response observed in 1/54 patients	[172]
Myc	Apto-253	20–150 mg/m^2^ i.v. once weekly		Phase Ia/b Clinical in relapsed/ refractory AML or high-risk AML patients (n = 18) (NCT02267863)	No DLTs or drug-related serious adverse events have been reported. 1 patient experienced a drug-related adverse event of grade 3 or greater (fatigue)	[173]
Bcl-2	Venetoclax	800 mg daily oral	-	Phase II clinical in relapsed/ refractory AML patients (n = 32) (NCT01994837)	Overall response rate was 19%; An additional 19% of patients exhibited partial bone marrow response and incomplete haematological recovery; Common adverse events included diarrhea, vomiting, nausea, febrile neutropenia and hypokalemia (grade 3/4)	[174]
		400, 800 or 1200 mg orally daily	Decitabine 20 mg/m^2^ day 1–5 i.v. or azacitidine 75 mg/m^2^ day 1–7 i.v. or s.c	Phase Ib clinical in treatment-naïve elderly AML patients (n = 145) (NCT02203773)	Common adverse events included constipation, diarrhea, nausea, febrile neutropenia, hypokalemia, fatigue, decreased white blood cell count and decreased appetite; CR + CRi was observed in 67% of patients, with a CR + CRi rate of 73% in the 400 mg venetoclax + hypomethylating agent cohort; Median duration of CR + CRi was 11.3 months, and median OS was 17.5 months	[175]
		400 mg orally daily	Azacitidine 75 mg/m^2^ s.c. or i.v. day 1–7	Phase III Clinical in treatment-naïve AML patients (n = 431) (NCT02993523)	Median OS in combination group was 14.7 months and 9.6 months in the control group (azacitidine alone); Incidence of complete remission was higher in the combination cohort than in control (36.7% vs. 17.9%, respectively), as was the CR + CRi (36.7% vs. 28.3%); The most common adverse events included nausea, thrombocytopenia, neutropenia and febrile neutropenia; Serious adverse events occurred more commonly in the combination cohort compared to the control (83% vs. 73%)	[176]
		100 mg day 1, 200 mg day 2, 400 mg day 3–28 orally	Decitabine 20 mg/m^2^ i.v. day 1–10	Phase II Clinical in treatment-naïve elderly AML patients (n = 168) (NCT034014193)	Overall response rate was 74%; the most common adverse events included febrile neutropenia (29%) and infections with grade 3/4 neutropenia (50%); 6 grade 5 adverse events, including infections with therapy-related grade 3/4 neutropenia and 1 case of renal failure unrelated to the study	[177]
		Not described	Fludarabine 30 mg/m^2^ i.v. day 2–6 + Ara-C 1.5 g/m^2^ i.v. day 2–6 + idarubicin 8 mg/m^2^ i.v. day 4–6 + filgrastim 5 mcg/kg s.c. day 1–7	Phase Ib/II Clinical in newly diagnosed and relapsed/ refractory AML patients (n = 45)	Overall response rate was 44/45 (98%); 89% (40/45) patients attained a composite complete response, including 37/40 patients (93%) who were measurable residual disease negative; common non-haematological adverse events included bacteremia, pneumonia, febrile neutropenia, and skin/soft tissue infections; Estimated 24 months EFS and OS were 64% and 76%, respectively	[178]
mTOR	Rapamycin	2 mg per os daily 14 days	-	Clinical study in refractory AML patients (n = 5)	No severe haematological or non-haematological side effects were observed; 2 patients achieved a leukocyte response, a prolonged response was seen in 1 patient; In the 3 other patients, blast counts remained stable or increased	[179]
	Temsirolimus	25 mg i.v. days 1, 8, 15	Clofarabine 20 mg/m^2^ i.v. day 1–5	Phase II clinical in older AML patients (n = 53)	Overall remission rate was 21%; median DFS was 3.5 months, and median OS was 4 months overall, and 9.1 months for responders; The most common non-haematological severe adverse events included transaminitis (11%), infection (48%), and febrile neutropenia (34%)	[180]
NF-κB	Bortezomib	1.5 mg/m^2^ s.c. or i.v. days 1, 4, 8, and 11	Pegylated liposomal doxorubicin 40 mg/m^2^ i.v. day 4, 21	Phase II relapsed/ refractory AML patients (n = 15)	10 patients completed 1 cycle of chemotherapy, 1 confirmed partial response with >50% blast reduction, no patient had a complete response; 2 additional patients had >50% blast reductions, including 1 achieving morphologic leukaemia free state but without count improvement; 5 patients exhibited progressive disease after cycle 1, in the remaining 5 patients who commenced cycle 2, no observed blast reductions persisted, and not patients exhibited improvements in marrow blasts with additional cycle	[181]
		1.3 mg/m^2^ s.c. days 1, 4, 8 and 11	Decitabine 20 mg/m^2^ i.v. day 1–10	Phase II clinical in treatment-naïve older AML patients (n = 163) (NCT01420926	No significant differences in OS or responses between the 2 treatment arms; CR + CRi was 39%, with median OS of 9.3 month; most common adverse event was febrile neutropenia	[182]
		1–1.3 mg/m^2^/d i.v. days 8, 15 and 22	Tipifarnib 2—mg bid orally day 1–21	Phase I clinical in AML and high-risk MDS patients (n = 11) (EudraCT 2006-004588-69; NTR 2959)	MTD was not reached; The most frequent side effect was myelosuppression; CR + CRi was observed in 3/11 (27%) patients, and stable disease in 3/11 (27%) patients; median OS was 449 days, and 2 patients were still alive at 4 and 4.3 years, including one patient with continuing complete response	[183]
Hypoxia	Evofosfamide (TH-302)	120–550 mg/m^2^ i.v. days 1–5	-	Phase I clinical in relapsed/ refractory AML (n = 39) and ALL (n = 9) patients (NCT01149915)	DLTs were grade 3 esophagitis, which was observed at 550 mg/m^2^ and grade 3 stomatitis and hyperbilirubinemia observed at 460 mg/m^2^; MTD for 30–60 min/day infusion was 460 mg/m^2^ and for continuous infusion was 300 mg/m^2^; combined overall response rate was 6%, with all responses seen in 30–60 min/day infusion arm	[184]
PD-1/PD-L1	Pidilizumab (CT-011)	0.2–6 mg/kg i.v.	-	Phase I clinical in AML (n = 8), non-hodgkins lymphoma (n = 4), chronic lymphocytic leukaemia (n = 3), hodgkins lymphoma (n = 1), MDS (n = 1) and multiple myeloma (n = 1) patients	No single MTD was defined; No change in the average percentage of blasts in the blood of AML patients, with the exception of 1 AML patient that exhibited a reduction in the number of peripheral blasts from 50% to 5%	[185]
	Nivolumab	3 mg/kg i.v. days 1, and 14	Azacitidine 75 mg/m_2_ i.v. or s.c. day 1–7	Phase II clinical in relapsed/ refractory AML patents (n = 70) (NCT02397720)	Overall response rate was 33%, and 6 (9%) patients had stable disease > 6 months; the overall response rate was 58% and 22% in hypomethylating agent-naïve and hypomethylating agent-pre-treated patients, respectively; grade 3/4 immune-related adverse events occurred in 11% patients	[186]
		3 mg/kg on day 24	Ara-C 1.5 g/m^2^ daily for 4 days and idarubicin 12 mg/m^2^ daily for 3 days	Phase II clinical in treatment-naïve AML (n = 42) and high-risk MDS (n = 2) patients (NCT02464657)	Median RFS of responders was 18.54 months; 6 patients had grade 3/4 adverse events including rash, colitis, transaminitis, cholecystitis and pancreatitis	[187]

AML—acute myeloid leukaemia; Ara-C—cytarabine; allo-HCT—allogenic haematopoietic cell transplant; CML—chronic myeloid leukaemia; CR + Cri—overall complete remission and complete remission with incomplete blood count recovery rate; DFS—disease free survival; DLT—dose limiting toxicities; MDS—myelodysplastic syndrome; OS—overall survival; RFS—relapse free survival; s.c.—subcutaneous; ‘-‘—not examined; p.o.—per oral.

## Data Availability

Not applicable.
